# Impact of prior immunotherapy on paclitaxel/bevacizumab in advanced non-squamous non-small cell lung cancer

**DOI:** 10.3389/fonc.2025.1675386

**Published:** 2025-10-27

**Authors:** Agathe Peyret, Clémentine Le Berrurier, Luc Heraudet, Tara Delon, Maéva Zysman, Mathieu Larroquette, Laura Leroy, Sophie Cousin, Charlotte Domblides

**Affiliations:** ^1^ Department of Medical Oncology, Hôpital Saint-André, Bordeaux University Hospital, Bordeaux, France; ^2^ Pulmonary Department, Pôle Cardio-thoracique, Hôpital Haut-Lévèque, Bordeaux University Hospital, Pessac, France; ^3^ Centre de Recherche cardio-thoracique, Bordeaux University, Bordeaux, France; ^4^ Department of Medical Oncology, Bergonié Institute, Bordeaux, France; ^5^ Laboratoire ImmunoConcEpt CNRS UMR5164, Bordeaux, France

**Keywords:** NSCLC, immunotherapy, chemotherapy, therapeutic sequence, anti-angiogenic treatment

## Abstract

Immunotherapy is becoming essential in the management of advanced non-small cell lung cancer (NSCLC); however, the best treatment sequence remains to be determined. Some data suggest that immunotherapy prior to chemotherapy with paclitaxel/bevacizumab (PB) appears to be an interesting option. Here, we study this sequence in a validation cohort from the Bordeaux Cancer Institute (France). We retrospectively included all patients with NSCLC diagnosed between 2015 and 2021, treated with PB directly after immunotherapy (CAI) or without prior exposure to immunotherapy (CWPI). The primary outcome was the time to treatment discontinuation (TTD), and the secondary endpoints were progression-free survival (PFS), overall survival (OS), overall response rate (ORR), and disease control rate (DCR). We included 121 patients: 60 in the CAI group and 61 in the CWPI group. Their characteristics were comparable, even for the main prognostic criteria. The median number of lines received was higher in the CAI group (4 vs. 2). There was a significant difference in TTD (HR = 0.55, 95% CI 0.34–0.72, p = 0.0002), PFS (HR = 0.60, 95% CI 0.41–0.87, p < 0.005), and ORR (58% versus 38%, p < 0.05) between the two groups, as well as a non-statistically significant trend toward better OS (7.40 vs. 3.70 months, HR = 0.76, 95% CI 0.52–1.10, p = 0.15). We found a significant difference in TTD, PFS, and ORR for PB after exposure to immunotherapy, with a trend toward better OS. This suggests that sequential treatment with immunotherapy followed by chemotherapy could be an interesting option after first-line treatment.

## 1 Introduction

Immunotherapy is an essential treatment in non-small cell lung cancer (NSCLC). Indeed, it improves overall survival (OS) as monotherapy or in combination with chemotherapy in the first-line setting (1–3).

Biologically, chemotherapy induces immunologic cell death, leading to the activation of the antitumor immune response through the release of neoantigens (4, 5). Beyond this direct effect, chemotherapy can also modify antitumor immune response by modulating the immunosuppressive landscape of the tumor microenvironment, potentiating the effect of immunotherapy. That is why, nowadays, the combination of chemotherapy and immunotherapy is one of the pivotal strategies for treating metastatic NSCLC.

However, some evidence suggests that a sequential treatment by immunotherapy prior to chemotherapy could enhance the chemotherapy duration response (6, 7). This sequence remains important for patients who receive immunotherapy alone as first-line treatment, for patients who progress after immunotherapy as a maintenance line, or for a rechallenge. However, such data are controversial, as some reports did not show any differences (8). Conversely, fewer studies based on small retrospective cohorts have shown a significant increase in the objective response rate, but not in treatment duration or overall survival (9).

Nevertheless, a recent study of our team (7) investigated the response to different chemotherapy protocols after immunotherapy and reported a better OS in the paclitaxel/bevacizumab (PB) cohort when PB was administered directly after immunotherapy.

Therefore, we aimed to confirm these results in a validation cohort of patients treated with PB just after an immunotherapy regimen.

## 2 Methods

### 2.1 Patients

We retrospectively included all consecutive patients with stage III/IV histologically proven NSCLC diagnosed between 1 October 2015 and 30 September 2021, without access to a local treatment, and treated at least with a second line at the Bordeaux Cancer Institute (France). Patients must have received at least one infusion of PB treatment directly after the immunotherapy regimen. Eligible patients could have undergone radiotherapy or surgery in the localized phase, with a recurrence after such therapies. For patients who had a rechallenge of PB later in their disease, we only considered their first exposure.

We excluded patients with oncogenic addiction (*EGFR*, *ALK*, and *ROS1*), as possible access to targeted therapy may lead to misinterpretation of overall survival data. We also excluded patients who had concomitant chemotherapy and immunotherapy as first-line treatment, as chemotherapy associated with immunotherapy could modify tumor response and patient prognosis. Finally, we also excluded patients treated for another cancer.

We collected the following data from medical records: age, gender, smoking status, histology, TNM stage according to the eighth edition, presence of liver and brain metastasis, PD-L1 status, and different treatment lines.

### 2.2 Clinical endpoints

The primary outcome was the time to treatment discontinuation (TTD) (time between the first exposure to PB and the date of treatment discontinuation or death).

The secondary endpoints were progression-free survival (PFS) (from the first day of PB until progression or death), OS (from the first day of PB until death or lost to follow-up), overall response rate (ORR) (complete or partial response), and disease control rate (DCR) (complete response, partial response, or stable disease).

### 2.3 Statistical analysis

A statistical test was performed using a type I error rate of 5%. Qualitative variables were described as numbers and percentages and compared using a χ^2^ test or corrected χ^2^ test for parametric variables, or a non-parametric Fisher’s exact test.

We described quantitative variables as median and first and third quartiles and compared them using a Student’s t-test (parametric) or a Wilcoxon (non-parametric) test. We described survival variables with survival probabilities. We used the Kaplan–Meier method to generate survival curves, and we used the log-rank test to compare survival endpoints.

We censored data at the time of last contact.

All statistical analyses were performed using GraphPad Prism v10. The study was conducted in accordance with French legislation and ethical codes. This work complied with the protection of personal health data and the protection of privacy with the framework of application provided by Article 65–2 of the amended Data Protection Act and the General Data Protection Regulation. The study was designed according to the STROBE guidelines (**Supplementary Methods**).

## 3 Results

### 3.1 Patients’ characteristics

**Figure 1:**
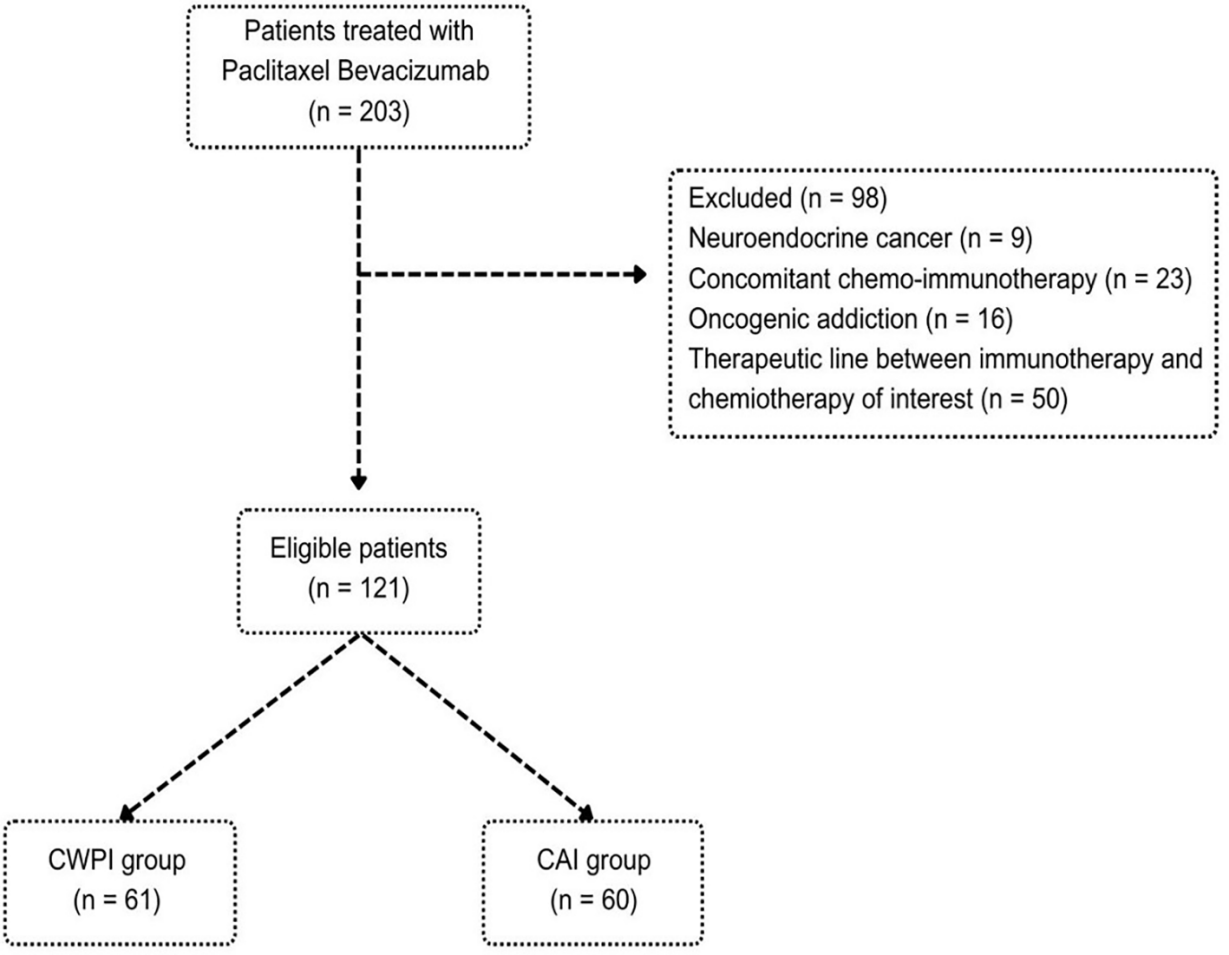
Flowchart for selecting our population of interest. CAI, chemotherapy after immunotherapy; CWPI, chemotherapy without previous immunotherapy.

Data from 203 patients who received the PB regimen were collected, but only 121 were included. Ninety-eight patients could not be included because of neuroendocrine cancer (n = 9), concomitant chemo-immunotherapy (n = 23), oncogenic addiction (n = 16), or a therapeutic line between immunotherapy and chemotherapy (n = 50) (**Figure 1**). Patients were divided into two cohorts: CWPI [chemotherapy without previous exposure to immune checkpoint inhibitor (ICI); n = 61] and CAI (chemotherapy after ICI; n = 60). Of these 121 patients, all received at least one infusion of PB.

The baseline characteristics of each group are summarized in **Table 1** and were comparable between the two groups. Briefly, the main histologic type was adenocarcinoma (97%) in both groups, as it is the main indication in France. Most patients had a preserved overall condition, with ECOG scores of 0–1 in 40% (CWPI) and 58% (CAI) of patients, and the difference was non-significant. At the first injection of PB, 37% of patients in both groups had brain metastases (p = 1), and there was a higher rate of liver metastasis in the CWPI group (32% vs. 18% in the CWPI and CAI groups, respectively), but the difference was non-significant.

**Table 1:** Demographic and disease characteristics of the patients at baseline.

Treatment	CWPI (n = 61)	CAI (n = 60)
Median age (Q1–Q3)	59 years (49–66)	62 years (52–67)
Gender		
Male Female	28 (45.9%) 33 (54%)	35 (58%) 25 (42%)
Smoking status		
Never Former Current Missing data	3 (4.9%) 27 (44.2%) 29 (47.5%) 2	7 (11%) 25 (42%) 28 (47%)
Histologic subtypes		
Adenocarcinoma Squamous Other	59 (96.7%) 0 (0%) 2 (3.27%)	58 (97%) 0 (0%) 2 (3%)
Line of chemotherapy of interest		
1 2 3 >3	2 (3.27%) 48 (78.6%) 12 (19.67%) 0	0 1 (1%) 46 (77%) 13 (22%)
Median number of lines received (range)	2 (2–3)	4 (2–6)
PD-L1 status		
<1% 1%–49% >50% Missing data	18 (29.5%) 1 (1.64%) 2 (3.27%) 40 (65.5%)	23 (38%) 12 (20%) 8 (14%) 17 (28%)
ECOG PS		
0–1 >1 Missing data	24 (39.3%) 24 (39.3%) 13 (21.3%)	35 (58%) 25 (42%) 0 (0%)
Type of ICI		
Nivolumab Pembrolizumab Atezolizumab Other		21 (35%) 12 (20%) 20 (33%) 7 (12%)
Brain metastasis	23 (37.7%)	22 (37%)
Liver metastasis	19 (31.1%)	11 (18%)

CAI, chemotherapy after immunotherapy; CWPI, chemotherapy without previous immunotherapy; PS, performance status; ICI, immune checkpoint inhibitor.

For the majority of patients, chemotherapy was received as a second (n = 48, 78.6%) (CWPI) or third line (n = 46, 77%) (CAI). PD-L1 status was non-interpretable, as there were several missing data. In the CAI group, the ICI was mainly received as a second line (n = 46, 77%) or a third line or more (n = 13, 22%). Only one patient received immunotherapy as first-line treatment. Nivolumab and atezolizumab were the most widely used ICIs [n = 21 (35%) and n = 20 (33%), respectively], followed by pembrolizumab (n = 12, 20%). The median number of lines received in total was two in the CWPI group and four in the CAI group. In the CAI group, patients mainly received a platinum-based doublet, and immunotherapy was only administered as a second-line or beyond (after several courses of chemotherapy). In the CWPI group, patients received a platinum-based doublet followed by chemotherapy, most often with paclitaxel and bevacizumab.

The median TTD under ICI treatment was 1.80 (1.10–3.40) months. The median PFS and OS for the entire cohort were 3.87 (1.84–6.82) months and 5.41 (2.46–11.43) months, respectively. The median time between the end of ICI and the beginning of PB was 1.1 months. There was no correlation between the time under immunotherapy and under ICI (p = 0.63).

### 3.2 CAI vs. CWPI

**Figure 2:**
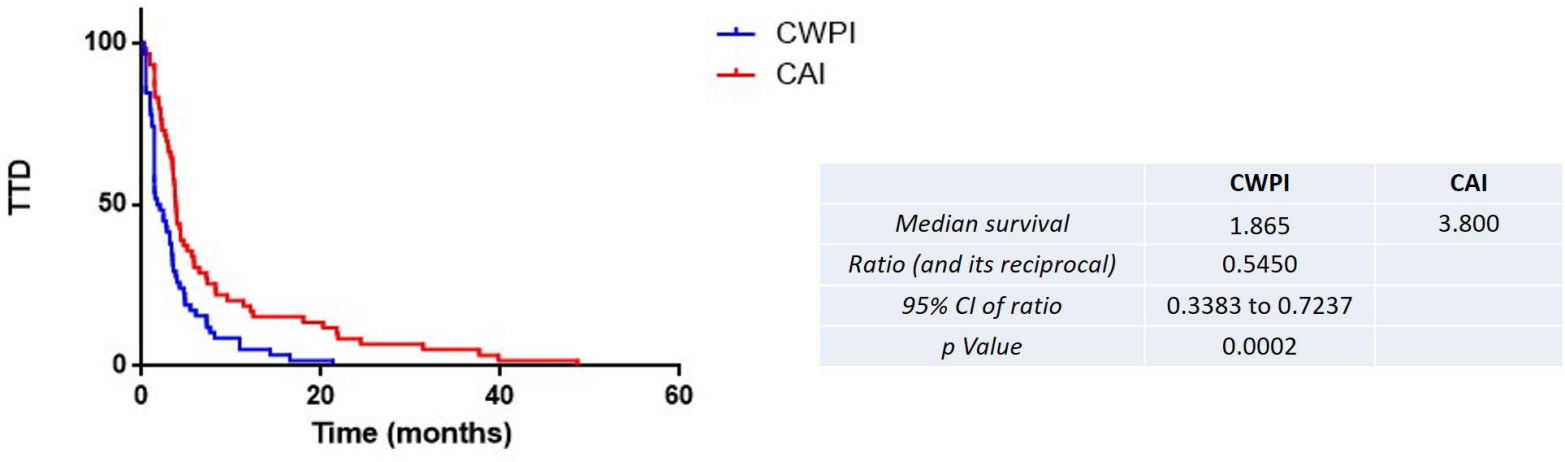
Kaplan–Meier estimates of the time to treatment discontinuation (TTF) according to the treatment arm. CAI, chemotherapy after immunotherapy; CWPI, chemotherapy without previous immunotherapy; HR, hazard ratio.

**Figure 3:**
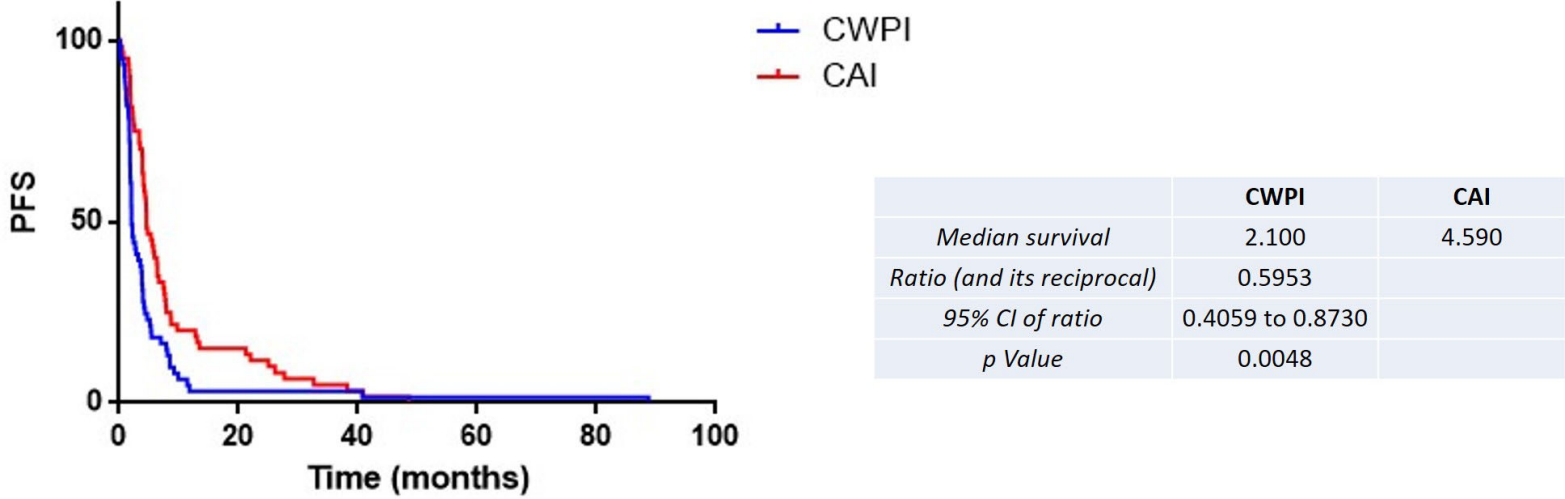
Kaplan–Meier estimates of the progression-free survival according to the treatment arm. CAI, chemotherapy after immunotherapy; CWPI, chemotherapy without previous immunotherapy; HR, hazard ratio.

**Figure 4:**
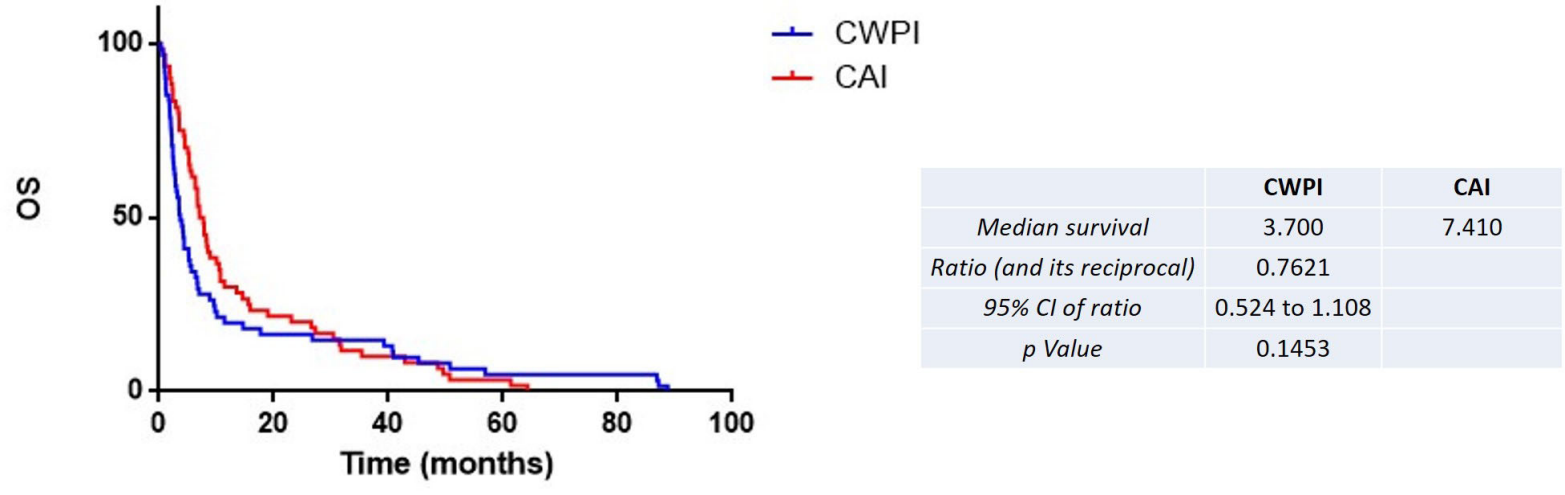
Kaplan–Meier estimates of the overall survival (OS) according to the treatment arm. CAI, chemotherapy after immunotherapy; CWPI, chemotherapy without previous immunotherapy; HR, hazard ratio.

The median TTD was higher for the CAI group (3.77 vs. 1.48 months for the CAI group vs. CWPI group, HR = 0.55, 95% CI 0.34–0.72, p = 0.0002) (**Figure 2**). The same results were found for PFS (4.60 vs. 2.10 months for the CAI group vs. CWPI group, HR = 0.60, 95% CI 0.41–0.87, p < 0.005) (**Figure 3**). Finally, there was a trend toward a better OS in the CAI group since survival was doubled, but this was not significant (7.40 vs. 3.70 months for the CAI group vs. CWPI group, HR = 0.76, 95% CI 0.52–1.11, p = 0.14) (**Figure 4**). Regarding the tumor response, 35 (58.3%) and 23 (37.7%) patients were responders, 11 (18.3%) and four (6.6%) had stable disease, and 14 (23.4%) and 34 (55.8%) progressed in the CAI and CWPI cohorts, respectively (**Table 2**). ORR and DCR were significantly better in the CAI group compared with the CWPI group (p < 0.05 and p < 0.001, respectively).

**Table 2:** Response rates in the CWPI and CAI groups.

	CWPI (N, %)	CAI (N, %)
Complete response (CR)	3 (4.9)	5 (8.3)
Partial response (PR)	20 (32.8)	30 (50.0)
Stable disease (SD)	4 (6.6)	11 (18.3)
Dissociated response (DR)	4 (6.6)	1 (1.7)
Progressive disease (PD)	30 (49.2)	13 (21.7)
ORR	38%	58%
DCR	44%	76%

CWPI, chemotherapy without previous immunotherapy; CAI, chemotherapy after immunotherapy; ORR, overall response rate; DCR, disease control rate.

Brain metastasis is one of the main prognostic factors in lung cancer. In this subgroup, survival was better in the CAI group [TTD 4.30 vs. 2.43 months (p = 0.10), PFS 5.90 vs. 2.07 months (p = 0.063), OS 6.30 vs. 3.48 months (p = 0.86), ORR 48% vs. 34.8% (p = 0.30), and DCR 65% vs. 43.5% (p = 0.06)]. In the group of patients with liver metastasis, survival was better in the CAI group for patients with liver metastases [TTD 3.51 vs. 1.38 months (p = 0.073), PFS 4.10 vs. 1.90 months (p = 0.006), OS 5.25 vs. 2.49 months (p = 0.046), ORR 45.5% vs. 21% (p = 0.15), and DCR 81.8% vs. 26% (p = 0.005)].

The benefit in PFS and TTD appeared to be greater in patients with an unfavorable performance status (PS). Indeed, for the PS > 1 group, PFS and TTD were better in the CAI group [PFS 3.40 vs. 1.85 months (p = 0.002) and TTD 2.25 vs. 1.38 months (p = 0.048), respectively], and there was a better trend for OS [5.15 vs. 2.59 months (p = 0.244)]. ORR was 42.3% in the CAI group and 33.3% in the CWPI group (p = 0.07).

Conversely, for PS 0–1, there was no difference in PFS, TTD, or OS [PFS 5.57 vs. 5.30 months (p = 0.95), TTD 4.07 vs. 4.46 months (p = 0.116), and OS 9.48 vs. 8.36 months (p = 0.744), respectively]. ORR was 67.6% in the CAI group and 54% in the CWPI group (p = 0.02). However, patients with poor status had a higher brain metastasis rate in the CAI group compared to the CWPI group (52% vs. 25%). Conversely, brain metastasis did not influence PFS and TTD for patients with a good PS. Finally, there were no differences regarding liver metastasis.

After failure of the studied strategy, 24 (39%) patients in the CAI group received subsequent therapy, with four undergoing ICI rechallenge (6.5%). In the CWPI group, 26 (43%) patients received another line after, including a crossover of 33% (n = 20) for ICI.

At the date of the last follow-up, nine (7.4%) patients were still alive: four (6.7%) in the CAI group and five (8.3%) in the CWPI group. Among them, five (4%) patients were still on treatment, and only one patient was still receiving maintenance bevacizumab in the CAI group.

## 4 Discussion

Immune checkpoint inhibitors modify oncologic treatments by significantly improving overall survival, sometimes with long-term responses, and preserving quality of life (1, 10).

Currently, there are several developments of concomitant combinations of chemotherapy and immunotherapy. However, while such therapeutic strategies are efficient in the first-line setting, many patients relapse, and there is an important need to specify further treatment lines and therapeutic sequence, concomitant or sequential. As a result, there are few therapeutic options, including resuming chemotherapy or, at a later stage, carrying out an immunotherapy rechallenge. Based on the ULTIMATE trial, the combination of paclitaxel and bevacizumab has become an interesting option in case of progression after first-line treatment, with an ORR of 22.5% (11). However, this protocol has not been evaluated after immunotherapy in this trial. Indeed, we based this study on our previous data showing a trend to an OS benefit of the PB protocol when administered after immunotherapy (7).

In this study, we found better TTD, PDSC, ORR, and DCR for patients treated with the PB protocol if administered after immunotherapy. We also observed a trend toward a better OS for these patients. Our results are slightly different from those obtained from another large cohort by our team, reporting only a trend toward an increase in OS (7). These differences could be explained by the larger number of patients included in this cohort, allowing the results to be refined. Furthermore, in this study, patients had fewer brain metastases than in Heraudet’s cohort (in the CAI group, 68% versus 38%), leading to better outcomes regarding the severe prognostic impact of brain metastasis (7). Finally, we observed a short TTD under the previous ICI, which did not prevent a response to chemotherapy, meaning that a poor response to immunotherapy is not a reason for not treating patients after immunotherapy with the PB protocol.

There is a biological rationale for combining chemotherapy with immunotherapy due to the reciprocal potentiation of both treatments. Indeed, immunotherapy sensitizes patients to chemotherapy by the induction of a priming of the immune system, improving its ability to react to chemotherapy-induced immunogenic cell death that releases neoantigens in the tumor microenvironment. In addition, chemotherapy could have immunomodulatory functions by resensitizing to further ICI therapy. Then, chemotherapy can directly modulate immune cells, increasing antitumor response while inhibiting some immunosuppressive cells. Indeed, paclitaxel favors the antitumor CD4^+^ T-cell phenotype and dendritic cell maturation, increasing antitumor immune response (12). Conversely, chemotherapy can resensitize patients to immunotherapy via its immunomodulatory effects. Clinical data therefore suggest that immunotherapy rechallenge after a course of chemotherapy is effective in patients who have already been treated with immunotherapy (13). Furthermore, anti-VEGF effect could be interesting to complete immunotherapy’s one, as this treatment inhibits VEGF, a growth factor that is a major contributor to angiogenesis and the immunosuppressive microenvironment (14, 15). The optimal timing of PD-1/PD-L1 blockade and chemotherapy, concomitant or sequential, remains unknown in the second line and beyond. Some preclinical data comparing both strategies in mouse models suggest an increased antitumor activity from chemotherapy when given before ICI, compared to concomitant chemo-immunotherapy (16).

In the literature, very few studies have assessed the efficacy of PB administered directly after ICI, with most of them studying the combination of docetaxel and ramucirumab. A larger study using PB, the French AVATAX trial, reported better outcomes for CAI in the subgroup analysis (17). In our study, survival was slightly lower than that in the AVATAX cohort, with consistent data, as there were also significant PFS improvement and a trend to a better OS. However, response rates were better for the CAI group (76.6% vs. 51%). In comparison, the ULTIMATE trial reported an ORR of 22.5% for PB without previous immunotherapy, meaning that the efficacy of chemotherapy associated with antiangiogenic treatment could be improved by previous immunotherapy (11, 17, 18). A trial by the IFCT group in France is currently ongoing, evaluating PB ± atezolizumab in non-squamous NSCLC.

In the subgroup analysis, we found better efficacy for the CAI group in patients with liver metastasis. Liver metastasis is known to release large amounts of VEGF, which is one of the most important proangiogenic molecules, and is also able to polarize immunosuppressive cells, such as M2-subtype macrophages (15). Furthermore, studies have shown that this type of metastasis had a lower infiltration of cytotoxic T lymphocytes, as well as NK and dendritic cells (19). For brain metastasis, we found a trend to better PFS and DCR in the CAI group. Other endpoints were non-significant, probably due to the small number of this subgroup, whereas there was a numerical increase in each endpoint. Regarding the brain, the immune landscape is very special, as several studies have reported a decreased infiltration of dendritic cells and T cells with Th1 antitumor polarization and a diminished extravasation of immune cells (20). Indeed, we hypothesize that even if immunotherapy does not have an important effect on metastasis, the efficacy of post-immunotherapy PB regimen could be improved through its direct and indirect immunomodulatory functions, increasing the immunotherapy efficacy in a sequential strategy. In addition, the time between the end of immunotherapy and the beginning of chemotherapy was only 1.1 months. This means that patients, while starting chemotherapy, were still under the effect of immunotherapy, as the lifetimes of the molecules are between 21 and 28 days.

A strength of our work is that it is a “real-life” study, with relevant clinical criteria, assessing the efficacy of PB administered after immunotherapy. Indeed, we chose TTD as the primary endpoint, as it is a pragmatic and more relevant endpoint to assess efficacy in real-world trials. Conversely, there are some limitations in our study. First, it was a monocentric and retrospective study, with missing data that prevented multivariate analysis. In addition, it should be noted that most controls were treated between 2016 and 2017, whereas those who received immunotherapy were treated between 2019 and 2020, a factor that could modify patient survival related to changes in recommended therapeutic strategies.

As a conclusion, this study found significant improvement of PFS, ORR, and TTD and a trend toward higher OS using PB directly after immunotherapy, where patients were heavily pretreated. This is an argument in favor of sequential treatments after the first line. However, further studies are needed to clarify the place of these sequential strategies in the context of first-line chemo-immunotherapy associations currently validated (4). Indeed, it could be relevant to study its efficacy in this setting, as the addition of concomitant chemotherapy could modify the immune contexture and the response to subsequent therapies.

## Data Availability

The original contributions presented in the study are included in the article/**Supplementary Material**. Further inquiries can be directed to the corresponding author.
