# Supplementary material for: Impact of prior immunotherapy on paclitaxel/bevacizumab in advanced non-squamous non-small cell lung cancer
Source: Front Oncol. 2025 Oct 27;15:1675386. doi: 10.3389/fonc.2025.1675386 (PMC12597745; doi:10.3389/fonc.2025.1675386)
Supplement: Supplementary file 1 [file DataSheet1.pdf]

ECOG Performance Status

These scales and criteria are used by doctors and researchers to assess how a patient's disease is progressing, assess how the disease affects the daily living abilities of the patient, and determine appropriate treatment and prognosis. They are included here for health care professionals to access.

ECOG PERFORMANCE STATUS*	
**Grade**	**ECOG**
0	Fully active, able to carry on all pre-disease performance without restriction
1	Restricted in physically strenuous activity but ambulatory and able to carry out work of a light or sedentary nature, e.g., light house work, office work
2	Ambulatory and capable of all selfcare but unable to carry out any work activities. Up and about more than 50% of waking hours
3	Capable of only limited selfcare, confined to bed or chair more than 50% of waking hours
4	Completely disabled. Cannot carry on any selfcare. Totally confined to bed or chair
5	Dead

* As published in Am. J. Clin. Oncol.:
Oken, M.M., Creech, R.H., Tormey, D.C., Horton, J., Davis, T.E., McFadden, E.T., Carbone, P.P.: Toxicity And Response Criteria Of The Eastern Cooperative Oncology Group. Am J Clin Oncol 5:649-655, 1982.
